# Ultrafast photonic micro-systems to manipulate hard X-rays at 300 picoseconds

**DOI:** 10.1038/s41467-019-09077-1

**Published:** 2019-03-11

**Authors:** Pice Chen, Il Woong Jung, Donald A. Walko, Zhilong Li, Ya Gao, Gopal K. Shenoy, Daniel López, Jin Wang

**Affiliations:** 1 Advanced Photon Source, Argonne National Laboratory, 9700 S. Cass Ave., Argonne, 60439 IL USA; 2 Center for Nanoscale Materials, Argonne National Laboratory, 9700 S. Cass Ave., Argonne, 60439 IL USA

## Abstract

Time-resolved and ultrafast hard X-ray imaging, scattering and spectroscopy are powerful tools for elucidating the temporal and spatial evolution of complexity in materials. However, their temporal resolution has been limited by the storage-ring timing patterns and X-ray pulse width at synchrotron sources. Here we demonstrate that dynamic X-ray optics based on micro-electro-mechanical-system resonators can manipulate hard X-ray pulses on time scales down to 300 ps, comparable to the X-ray pulse width from typical synchrotron sources. This is achieved by timing the resonators with the storage ring to diffract X-ray pulses through the narrow Bragg peak of the single-crystalline material. Angular velocities exceeding 10^7^ degrees s^−1^ are reached while maintaining the maximum linear velocity well below the sonic speed and material breakdown limit. As the time scale of the devices shortens, the devices promise to spatially disperse the temporal width of X-rays, thus generating a temporal resolution below the pulse-width limit.

## Abstract (web summary)

It is desirable to improve spatiotemporal control of light generated by synchrotron user facilities or table-top X-ray sources. Here the authors demonstrate manipulation of hard X-rays using microelectro mechanical systems (MEMS) oscillators on timescales of 300 ps, approaching the synchrotron pulse width.

## Introduction

Materials with nanoscopic-to-mesoscopic structures have taken center stage in advancing science and technology. There have been major efforts in establishing the structure-function relationship of materials on these length scales employing a variety of physical and chemical probes, and hard X-ray tools have played an important role to this effort^1–6^. However, a deeper understanding of energy conversion, storage, transmission, and utilization requires a complete mapping of the spatiotemporal behavior of relevant processes in, for example, solar and thermoelectric conversion, fuels cells and batteries, and efficient and clean combustion^7–11^. These processes include carrier dynamics, phonon transport, ionic conduction, multicomponent diffusion, phase transformation, interfacial diffusion, multiphase fluid flow, strain propagation, and soot formation on the temporal scales of microseconds and less^12–18^. Spatiotemporal X-ray probes with similar time resolution and spatial resolution—from picometers to mesoscopic scales—are essential to meeting this challenge. While X-ray free-electron lasers (XFELs) with a femtosecond pulse width are extremely effective in probing dynamics on ultrashort time scales, synchrotron-based X-ray sources are well suited for revealing the spatiotemporal evolution of mesoscopic details in materials. However, temporal resolution at synchrotron sources is generally limited by the X-ray pulse duration in the range of 10–100 s of picoseconds. Accessing shorter time scales, for example a few picoseconds, requires complex and costly modification of the storage ring^19–23^ at the expense of other source characteristics such as intensity and brightness.

On the other hand, photonic devices based on microelectromechanical systems (MEMS) technology have been implemented in a wide range of applications and scientific research^24–26^. The ability to manipulate light dynamically in a compact package is highly desirable in many scientific and technological applications. In addition, favorable scaling laws for miniaturization result in capabilities not possible with macro-scale devices. In the MEMS photonics community, the wavelengths of interest have been mainly in the visible to infrared regions for a wide range of imaging and telecommunication applications^27,28^. Previously, we showed that a MEMS oscillator, asynchronous to the X-ray source, can create and preserve the spatial, temporal, and spectral correlation of the X-rays on a time scale of several nanoseconds^29^.

In this work, we demonstrate that a MEMS-based X-ray dynamic optics, oscillating with a frequency matched to a synchrotron storage ring with a 1.1-km circumference, can control and manipulate hard X-ray pulses significantly below one nanosecond at 300 ps. With this exceptional time scale, we are now one step closer to achieving pulse streaking and pulse slicing that would allow us to access information at a sub-pulse temporal scale.

## Results

### MEMS devices as dynamic X-ray optics

**Fig. 1:**
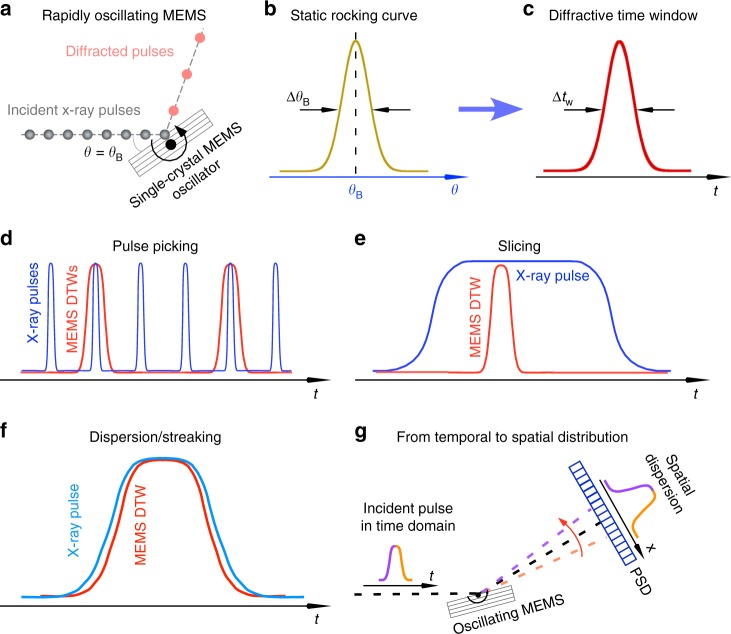
Manipulation of hard X-ray pulses using a microelectromechanical-system (MEMS)-based oscillator. **a** Schematic of a rapidly oscillating single-crystal micromirror in a torsional MEMS device that diffracts monochromatic X-rays at its Bragg angle. **b** Static crystal rocking curve around Bragg angle *θ*_B_ with a full-width-at-half-maximum (FWHM) of Δ*θ*_B_, typically several milli-degrees. **c** Around the instance that the single-crystal element rotates through the Bragg angle, the crystal rocking curve converts to a temporally dispersed diffractive time window (DTW) with a FWHM of Δ*t*_w_. **d** When the DTW width is much wider than the X-ray pulse, but narrower than the pulse-to-pulse spacing, the MEMS can be utilized as an ultrafast pulse-picking device. **e** When the DTW is narrower than the X-ray pulse, the device creates X-ray pulses shorter than the incident pulse width in a form of pulse slicing in the time domain. **f** Dispersion/streaking of the X-ray pulse is possible, when the MEMS DTW is close to the incoming pulse width. **g** In the dispersion/streaking mode using a position-sensitive detector (PSD), the  oscillating MEMS converts the X-ray pulse in the time domain to a spatially dispersed signal that contains time-resolved, sub-pulse information

The concept of using a MEMS device in the X-ray wavelength range as a dynamic diffractive optics for a monochromatic beam is shown schematically in Fig. 1. A thin, single-crystal silicon MEMS can diffract or transmit X-rays just by a change in its orientation relative to the incident X-ray beam (Fig. 1a). When the Bragg condition is fulfilled, the diffractive beam intensity as a function of the incident angle, *θ*, can be described by a rocking curve, illustrated in Fig. 1b, around the Bragg angle, *θ*_B_. As the MEMS crystal rapidly oscillates in the vicinity of *θ*_B_, a diffractive time window (DTW, as shown in Fig. 1c) opens during that time period. There are three schemes for utilization of MEMS devices: First, when the DTW is wider than the individual synchrotron X-ray pulses, but narrower than the pulse interval, the MEMS device can be employed as an X-ray pulse-picking chopper, as shown in Fig. 1d. Second, if the DTW is narrower than the X-ray pulse width (as shown in Fig. 1e), the MEMS device will generate X-ray pulses shorter than the incident pulses, which enables time-resolved experiments to be performed with a temporal resolution higher than that given by the incident pulse duration. Parenthetically,  this scheme also applies to continuous X-ray beams such as lab sources. Third, when the DTW is comparable to or slightly narrower than the width of the X-ray pulse (as seen in Fig. 1f), an X-ray streaking theme can be envisioned, completely in the optical domain, without sacrificing the detection efficiency, a problem suffered by other photonic devices such as X-ray streak cameras^30^. The conversion from X-ray pulse in the time domain to a streaked signal in the spatial domain is illustrated in Fig. 1g, where a high-resolution, position-sensitive detector accomplishes the conversion. This could lead to information in the single-pulse duration with sub-pulse temporal resolution. To make possible the applications of MEMS devices as dynamic X-ray optics, the MEMS DTW has to be sufficiently narrow. A high-frequency device is essential for pulse-picking, while a minimum DTW is more critical for pulse-slicing and streaking applications.

Since MEMS devices are based on single-crystal silicon (see Methods), X-ray diffraction occurs at the Bragg angle, *θ*_B_, at which the incident X-rays satisfy the Bragg condition. Due to the dynamical diffraction process^31^, the angular width of the diffraction condition is not zero, but has a finite value (Δ*θ*_B_, or rocking curve width), as illustrated in Fig. 1b. As the MEMS device oscillates around *θ*_B_ (Fig. 1a), the single-crystal element will diffract the X-rays for the short amount of time in which the Bragg condition is satisfied, and the element will transmit and absorb the X-rays over the rest of the cycle. The oscillatory device transforms the static rocking curve into a dynamic one in the time domain, i.e., DTW (Fig. 1c). The maximum angular speed of the MEMS device determines the width of the DTW over which the Bragg condition is fulfilled. For use as a monochromatic X-ray optic, the width of the DTW, Δ*t*_w_, is given by^29^

1 $$\Delta t_{\mathrm{w}} = \frac{{\Delta \theta _{\mathrm{B}}}}{{2\pi f\alpha }},$$

where *f* and *α* are the MEMS oscillation frequency and amplitude, respectively. In order to interact with X-ray pulses while preserving their spatiotemporal correlation, a MEMS device must perform as an X-ray diffractive element with the highest reflectivity while maintaining this performance at high speeds without introducing any distortion to the incident X-ray wavefront^29^.

### Tuning the MEMS resonant frequency to match the storage-ring frequency

To be a dynamic optics for pulsed X-rays in an efficient way, the oscillation must be in synchrony or frequency-matched with the X-ray source. Since MEMS resonators with a quality factor (Q) exceeding 10^3^ have an extremely narrow resonance bandwidth, it is virtually impossible for an as-fabricated MEMS device to have a resonant frequency that coincides with the storage-ring frequency. An asynchronous device cannot be an effective X-ray optics at a light source that produces periodic pulses. In order to tune the frequency of the MEMS devices to be commensurate with the frequency of the synchrotron ring, we developed a highly precise frequency trimming process based on focused ion beam (FIB) techniques (see Methods and Supplementary Note 1). The tuning process started with an as-fabricated MEMS with a resonance frequency of 65.8 kHz, about 2 kHz lower than the desired frequency of 67.889 kHz (one-fourth of the Advanced Photon Source [APS] operation frequency of P0 = 271.555 kHz).

**Fig. 2:**
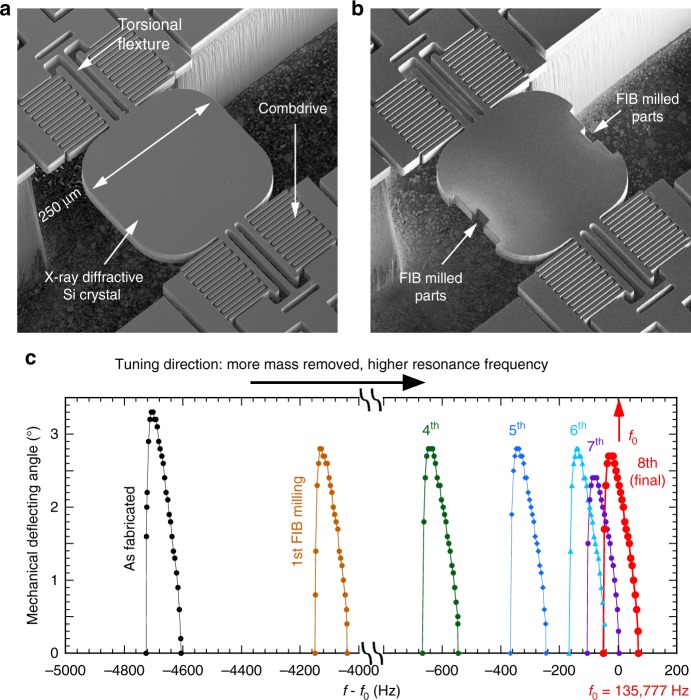
Tuning the resonance frequency of a MEMS oscillator using focused ion beam (FIB). **a** **b** Scanning electron microscopy images of the MEMS device as fabricated and after multiple rounds of FIB micromachining, respectively. Note these FIB machined devices still have the phosphorous dopant-induced strained layers, which was reported in our previous work^29^ (more detail in the Supplementary Note 2). **c** Tuning curves of the MEMS device measured with 45-V (peak-to-valley) square pulses after each FIB micromachining. Note that the frequency denoted in the X-axis is the frequency of excitation voltage signal, or twice the resonance frequency of MEMS devices. A total of eight micromachining processes were performed to tune the device to 135.777 kHz (P0/2)

As can be seen in Fig. 2a, the MEMS device we used is a torsional oscillating silicon crystal that is 25 µm in thickness and 250 × 250 µm in area, actuated with in-plane comb-drive actuators. The sinusoidal oscillation of the silicon crystal is excited by a square-wave voltage signal with a frequency twice that of the resonant frequency of MEMS devices^32^ at 135.777 kHz, or P0/2. Tuning the resonance frequency of the device was performed using the FIB tool to remove mass from the oscillating crystal, thus reducing its moment of inertia and increasing its resonance frequency (Fig. 2b). Removal of a 5 × 5 × 25-µm^3^ volume of the crystal results in an 80-Hz increase in the driving frequency of applied excitation voltage. The tuning curves (shown in Fig. 2c as a function of the driving frequency) have the characteristic waveform of a nonlinear resonator, with a sharp edge on the low-frequency side. The peak frequency of the tuning curves showed linear shifts with respect to removed volume at the outer edges of the silicon crystal (Supplementary Note 1 and Supplementary Figure 1). With careful calculations and control of the FIB micromachining, the frequency response of the device (at a 45-V driving voltage) was shifted to the range from 135.75 to 135.81 kHz, which overlaps with the desired frequency of 135.777 kHz or P0/2 (Fig. 2c). Hence, after the tuning process, this group of MEMS are denoted as P0/2 devices. We also note here that the method of FIB fine-tuning, together with the design flexibility of MEMS devices, allows us to deploy them at different synchrotron facilities worldwide (see Methods).

### Reducing diffractive time window by increasing excitation voltages

Per Eq. 1, to achieve a narrow window with a MEMS device of fixed resonance frequency, the most direct and effective method is to increase the oscillation amplitude, *α*, by applying a higher excitation voltage. This promises to provide a flexible DTW width from a few nanoseconds (as demonstrated previously^29^) to sub-nanosecond, as described below.

**Fig. 3:**
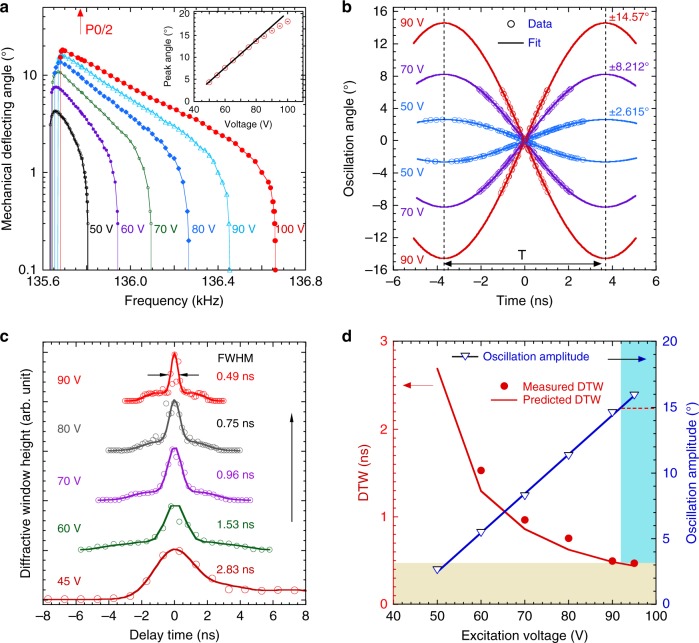
Reaching sub-nanosecond diffractive time window by applying high excitation voltage. **a** Tuning curves of the P0/2 MEMS at excitation voltages from 50 to 100 V in the vicinity of P0/2, or half the Advanced Photon Source storage-ring frequency. Inset to **a** shows the peak amplitude as a function of the excitation voltage. **b** Precise measurement of oscillation amplitude using the X-ray pulses while the MEMS device is excited exactly at a frequency of P0/2. We note that the delay time covered only a fraction of the MEMS oscillation period near null delay time, where the oscillation amplitude measurement is extremely sensitive and more accurate than any optical and electrical methods available to date. T is the oscillation period equal to 2/P0, or 7.365 μs. **c** DTW measured by 8-keV X-ray time-delay scans when the excitation voltages increased from 60 to 90 V, at which time the DTW width drops to below 0.5 ns. The curve denoted as 45 V is from an as-fabricated device in our previous work^29^ that is not frequency-matched to X-ray pulses. The arrow pointing upward marks the direction of increasing oscillation amplitude. **d** Comparison between the expected and measured DTW as a function of the excitation voltage at 135.777 kHz. The expected DTW is calculated using Eq. 1 and the amplitude values from **b**, which have a linear relationship with the excitation voltage, shown as the blue symbols (data) and line (linear fit). The shaded areas indicate sub-nanosecond DTW where it becomes impractical to use a higher voltage to achieve narrower DTW in P0/2 MEMS devices

After FIB micromachining, and at a 45-V excitation voltage, the target frequency (P0/2) falls to almost the middle of the tuning curve. This ensures that the MEMS device can oscillate with an amplitude close to the peak values over a wide range of excitation voltage. In Fig. 3a, the tuning curves of the device are shown as the excitation voltage is varied from 50 to 100 V, above its onset voltage of about 40 V. Note that the mechanical deflecting angle is the physical angle that the MEMS crystal element oscillates around its flexure from the rest position, which is one-fourth of the MEMS optical scan angle, the widely used figure-of-merit in the MEMS scanner community. Around the onset voltage, a typical vertical comb-drive MEMS actuator has its oscillation angle proportional to the square of voltage^24^. Our measurement here covers the medium-to-high voltage region, where the peak amplitude increases almost linearly up to about 80 V, and deviates from the straight line at higher voltages (inset to Fig. 3a). The MEMS oscillation amplitude at P0/2 is measured precisely by the APS X-ray pulse (using 8-keV photons), as shown in Fig. 3b. The measurement was performed with closely packed synchrotron pulses (324-bunch mode at the APS with a bunch interval of 11.37 ns), which is discussed in detail in Methods and Supplementary Note 3. The angle vs. time plots during one cycle of the 7.365-µs oscillation reveal extremely precise oscillation amplitudes of 14.57° at 90 V, indicating the responsiveness of the MEMS oscillation to the excitation voltage. Parenthetically, this angle corresponds to an astonishing 58.3° optical scan angle at 135.777 kHz, while the peak optical scan angle can be close to 80°, as indicated by Fig. 3a.

With the frequency-commensurate MEMS device, the determination of the DTW can be efficiently done by means of delay scans with a single, 100-ps-wide synchrotron X-ray pulse, given that the pulse width is narrower than the DTW. The description of the delay scan is given in Methods and Supplementary Note 4. Briefly, by varying the phase of the device oscillation with respect to a fixed timing signal from the accelerator, an X-ray pulse samples through the Bragg peak in controlled delay steps so that the window profile is measured with an accuracy of 20 ps. Such delay scans can also be understood as the creation of the dynamic rocking curve of the MEMS element as illustrated in Fig. 1c. With the increase in oscillation amplitude by increasing excitation voltage, the DTW widths decrease steadily, from several ns to just below 0.5 ns at 90 V, reaching almost an order of magnitude decrease (Fig. 3c). This remarkable result is summarized in Fig. 3d, where the plots show the measured DTW widths match the values predicted by Eq. 1 extremely well, down to 0.49 ns at the highest voltage. We demonstrated that the MEMS element can effectively manipulate hard X-ray pulses on a 500-ps time scale, well below the shortest bunch interval at any synchrotron source, which is typically between 2 and 10 ns.

To evaluate the quality of the diffracted X-ray pulses by the MEMS element, we measured the diffracted beam spatial profiles in the diffraction plane when the MEMS devices were static or oscillating, as they are compared with the incident beam spatial profile. The result is documented in detail in Supplementary Note 5. We report here that the quality of the spatial profile of the diffracted beam remains high. While the diffraction efficiency is about 95% (with over 10 sampling points), the dynamic diffracted beam was broadened spatially by only about 20% compared to when the MEMS element is static (off). This degradation of diffracted beam quality is expected to be mitigated by the MEMS without the surface doping produced in future dedicated MEMS fabrication runs. In addition, with monochromatic X-ray beams, beam heating effect on the diffracted beam quality is negligible, as discussed in detail in Supplementary Note 6.

In Fig. 3d, the MEMS deflection angle at 135.777 kHz increases linearly with the excitation voltage. Using a higher excitation voltage alone to achieve a narrow DTW, however, has its limitations. With planar comb drives, the maximum oscillation amplitude is set by a pull-in phenomenon where the rate of electrostatic force starts to exceed the mechanical restoring torque, leading to unstable oscillation. In the pull-in-free region, we can estimate the maximum angle based on the geometry of the MEMS device^33^. In our case, the maximum angle is 20.3°. As shown in Fig. 3, the amplitude has already exceeded 15° at 90 V (blue shaded area in Fig. 3d), hence there is little room for further improvement. The calculated limit agrees well with the experiment as the measured amplitude saturates around 20° above 100 V. We have also estimated the mechanical limit of our MEMS device where a large oscillation amplitude leads to fracture failure of the device (Supplementary Note 7). The mechanical limit is about 40° (substantially beyond our measurement range) thanks to the excellent mechanical properties of silicon. In addition to a limited oscillation amplitude, at the high excitation voltages the tuning curve becomes significantly broadened, indicating lower Q factors (Fig. 3a) and higher energy dissipation. Therefore, it becomes impractical to rely on exciting the MEMS using a higher voltage (power) to achieve even narrower DTW (yellow shaded area in Fig. 3d).

As seen in Eq. 1, the most challenging technical requirements in developing dynamic X-ray optics are simultaneously achieving large-amplitude and high-frequency operation using the MEMS torsional oscillators, as well as retaining the X-ray diffraction quality of the MEMS crystal. MEMS devices have demonstrated frequencies of 100 MHz to 10 GHz in timing applications^34^ where the oscillation amplitude is not a part of the design merit. On the other hand, large deflecting angular amplitude devices have been developed for displays^28,35^, optical scanners^36,37^, and other beam steering applications. Most of these applications are limited by the requirements of other essential parameters, which do not require high oscillation frequency. Our devices require simultaneous optimization of both parameters: frequency and amplitude of the torsional devices. In addition to reducing DTW of the X-ray MEMS devices, higher resonant frequency, *f*, also improves the efficiency of X-ray delivery since the synchronized high-frequency devices have a greater duty cycle.

### Higher-frequency MEMS oscillators operated in vacuum environment

**Fig. 4:**
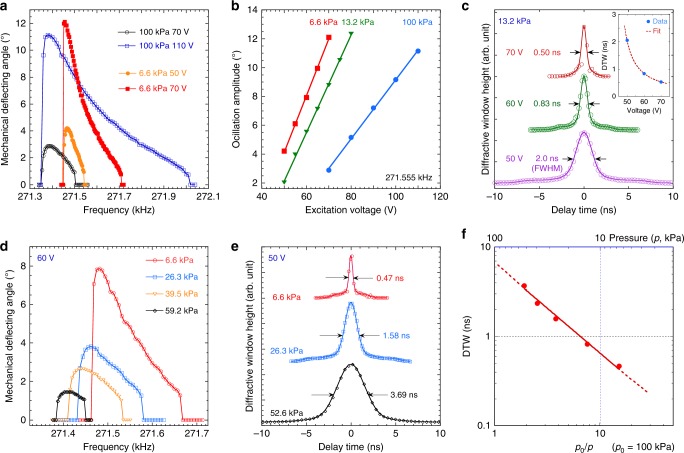
Performance of higher-frequency (P0) MEMS resonators operated in vacuum. **a** Tuning curves of the P0 device at atmospheric pressure (100 kPa) environment at the onset voltage of 70 V (black) and at a very high voltage of 110 V (blue), and in a reduced pressure of 6.6 kPa at 50 V (orange) and 70 V (red). At 6.6 kPa, similar oscillation amplitudes can be achieved at much lower voltages and the Q factor of the oscillation is also improved as the tuning curves become much narrower. **b** Dependence of the oscillation amplitude on excitation voltage at 100 kPa and reduced pressures. Note that an amplitude of 10° can be achieved at lower voltages of 70 V (13.2 kPa) and 60 V (6.6 kPa). **c** DTW measurement using delay time scans at excitation voltage ranging from 50 to 70 V at a reduced pressure of 13.2 kPa. The DTW width reaches 0.5 ns at a moderate voltage of 70 V. The dependence of the DTW on the excitation voltage is shown in the inset to the figure, where the fit (line) assumes a linear relationship between the oscillation amplitude and the voltage. **d** Tuning curves of the P0 device operated in a pressure range from 59.2 kPa down to 6.6 kPa at an excitation voltage 60 V. **e** Time delay scans of the high-frequency device at 52.6, 26.3, and 6.6 kPa at 60 V. The DTW width reaches below 0.5 ns at 6.6 kPa even at this modest excitation voltage. **f** Dependence of DTW on the environmental pressure at 60 V. The DTW  width (circle) decreases almost one order of magnitude from 3.69 ns to 0.47 ns as the pressure drops from 52.6 to 6.6 kPa

We designed MEMS devices with higher frequency and addressed the challenge to maintain a large oscillation amplitude. These MEMS devices, after FIB-based tuning, operate at the higher frequency of 271.555 kHz, the same as the APS storage-ring frequency, P0. We denote these as P0 devices. However, when operating in air, they have a much higher onset excitation voltage of 70 V, compared to lower-frequency devices (e.g., 40 V for the P0/2 devices). To obtain an oscillation amplitude above 10°, the excitation voltage must be as high as 110 V (as shown in Fig. 4a) with very high-level power consumption. The requirement of higher excitation voltage is due to the increased stiffness of the torsional flexure^24^ and significant fluid dynamic damping^38^ by the presence of air surrounding the rapidly oscillating devices whose angular velocity is close to 10^7^ degrees s^−1^. The interaction of the surrounding fluid (air) with the vibrating structure leads to energy dissipation, adversely affecting the oscillation amplitude and Q factor^38^.

In-vacuum operation greatly reduces the required excitation voltage of MEMS devices. Even in a moderate vacuum environment of 6.6 kPa, as shown Fig. 4a, the P0 device starts to oscillate at a much lower voltage of 40 V. At 50 V, the maximum amplitude is above 4°. When the excitation voltage was increased to 70 V (the same as the onset voltage in air, 100 kPa), the amplitude reached 12°, corresponding to a maximum angular velocity of 1.03 × 10^7^ degrees s^−1^. In addition, the Q factor, estimated from tuning curves with similar maximum amplitudes, was improved by a factor of 2.5 in the reduced pressure of 6.6 kPa. The dependence of the amplitude on excitation voltage at atmospheric (100 kPa) and reduced pressure conditions (13.2 and 6.6 kPa) is summarized in Fig. 4b. At all pressures, the relationship is simply linear in the measured range, but the 6.6-kPa data have a slope almost twice as much as the 100-kPa case. This increase of sensitivity to the excitation voltage at lower pressures can be explained by the improvement of Q factors due to lower energy dissipation. At 13.2 kPa, the voltage dependence of the MEMS diffractive window was measured again using the delay scan method described in Methods and Supplementary Note 4. When the excitation voltage increased from 50 to 70 V, the DTW steadily narrowed, from 2.0 ns at 50 V to 0.5 ns at 70 V (Fig. 4c). The decreasing DTW widths can be fit with a simple inverse relationship with the voltage shown in the inset to Fig. 4c, confirming the linear dependence of the amplitude on the excitation voltage. We note again that even in reduced pressure conditions, simply driving up the voltage will have a lessening effect on the reduction of DTW at higher voltages. Therefore, it is important for us to explore the effect of reduced pressure on the MEMS operational characteristics.

The reduction of fluid damping at lower operating pressure can increase the MEMS oscillation amplitude dramatically. As shown in the tuning curves in Fig. 4d, at a moderate excitation voltage of 60 V, the onset pressure is about 59.2 kPa, at which the amplitude was 1.45°. The tuning curve spans only a 50-Hz frequency range. Further reduction of the pressure to 6.6 kPa resulted in a much higher amplitude of 7.86°. Note that the tuning curves shift to higher frequencies when pressure decreases, indicating that the operating pressure can be another parameter to precisely tune the resonant frequency of the MEMS. The increase of the amplitude is also observed with the DTW measurement by the X-ray delay scans shown in Fig. 4e. The FWHM of the DTW decreased from 3.69 ns to 0.47 ns over the pressure drop. In Fig. 4f, we show that the DTW drop maintains a precise power-law relationship with the pressure drop in this coarse vacuum condition. Extrapolation of this relationship suggests a further reduction of DTW is possible at even lower pressures.

### Achieving a diffractive time window of 300 ps

**Fig. 5:**
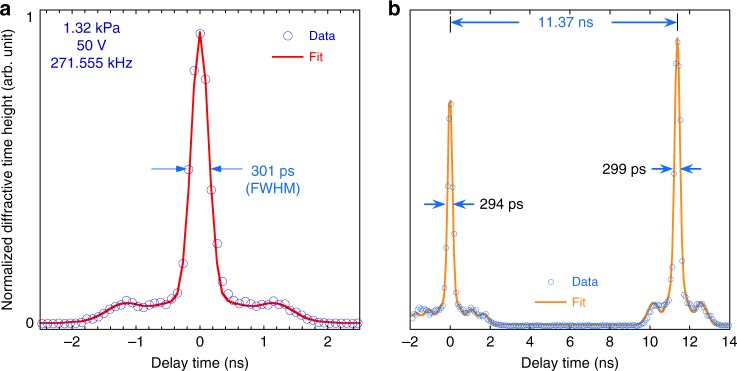
Demonstration of 300-ps diffractive time window. **a** Delay scan of a single X-ray pulse showing a 301-ps DTW. **b** Time-delay scan covering two consecutive pulses in the Advanced Photon Source 324-bunch mode when the two X-ray pulses are 11.37 ns apart. The scan clearly demonstrates DTW widths of 300 ps over the 16-ns delay time, measured in a time span of ca. 235 s

With a similar P0 device, we found that at an even lower excitation voltage of 50 V, and in a 1.32-kPa pressure environment, the device’s DTW width (FWHM) reduced to 301 ± 6 ps (Fig. 5a). The scan was performed using an APS X-ray pulse from the 324-bunch timing mode, where 324 singlets, each with nominally the same charge, are uniformly spaced in the storage ring. The spacing between the singlets is 11.37 ns with a normal pulse width of 51 ps FWHM (the storage-ring operation modes available at https://ops.aps.anl.gov/SRparameters/node5.html). The spacing between the DTW peaks was measured to be 11.37 ± 0.004 ns (Fig. 5b), agreeing well with the storage-ring parameter. Over this relatively long delay scan (covering a 16-ns range), which took ca.  235 s in real time to accomplish, the DTW widths determined by scanning through the two adjacent pulses were 294 and 299 ps, respectively. The pulse spacing can be utilized as an external clock to calibrate the delay time if there is timing jitter and/or phase drift in the MEMS operation. In this case, no correction or calibration was needed to obtain the X-ray pulse spacing and the DTW widths. The other observation is that the diffractive window heights revealed the relative charge variation in the bunches. Although all 324-bunch charges are nominally the same in the storage ring, the actual charge is measured to vary by up to 50%, as determined by the MEMS delay scans.

The P0 device has a static rocking curve width of about 3.7 mdeg (shown in Supplementary Note 2). The 300-ps DTW can be generated only by an angular velocity of 1.2 × 10^7^ degree s^−1^. This value is achieved by a device associated with a maximum linear velocity of only 25 m s^−1^ (less than one tenth the speed of sound in air) at the MEMS edges farthest from the flexure (125 µm), a unique advantage offered by the miniaturized and dynamic X-ray optics.

## Discussion

We note that the current 300-ps DTW is limited by the electronic noise in the driving circuits and the coarsely controlled environment where temperature and pressure around the devices can fluctuate. Fundamentally, and also remarkably, the specifications achieved in this work are far from their limits and still have room for further improvement. The maximum amplitude of the device can be ultimately limited by several factors, including the yield strength of silicon^39^, fluidic damping^38^, and instability due to large forces vs. flexure strength^40^. For the pulse pick theme (Fig. 1d), we speculate that the physical limitation of the oscillation frequency of the microscopic (100 s µm) MEMS devices could be on the order of 10 MHz. To achieve synchrotron pulse slicing (Fig. 1e) on a 3-ps time scale, a MEMS device would need to run at a 50-MHz frequency and 15° amplitude, which has not been demonstrated. However, MEMS devices capable of dispersing/streaking should be attainable because future devices can run at a frequency of a few MHz while retaining ca. 5° oscillation amplitude, which is sufficient to improve the DTW to 100 ps and below. The resulting improvements will lead to full utilization of the advantages that come from ultra-bright X-ray sources for science in the time domain^41^. These sources are often associated with very high bunch repetition rates (100–500 MHz) and a stretched bunch width (approaching 200-ps FWHM) to avoid the space charging effect for improving emittance^41^. Therefore, there is great potential for a higher-frequency MEMS, with time windows comparable to or narrower than the temporal width of the X-ray pulse itself, to be operated in an X-ray dispersion/streaking theme just in the X-ray optical domain, as illustrated in Fig. 1f, g. This will yield time-resolved information on sub-bunch time scales to facilitate time domain science without fundamentally altering the storage ring.

Thus far, there have been several electron deflecting methods utilized in the storage ring to generate deflected X-ray pulses that differ from unperturbed pulse trains for time-resolved experiments. The techniques employed kicker magnets^42^ or a quasi-resonant excitation of incoherent betatron oscillations^43^. Both methods delivered selected X-ray pulses with similar spectral distributions as undeflected X-rays. The picked X-ray pulses propagate roughly along the unperturbed main beam, so that it only requires minimum modifications in the beamline instrument to use the picked pulses. Also, the picked pulses have similar photon wavelength spectral distribution (with a significant reduction in brilliance) so that the beamline can retain white- and pink-beam experiment capabilities. However, these electron-deflection schemes were only realized at low and medium-energy sources. With the MEMS scheme, pulse-picking works in monochromatic beam mode and the spectral brilliance is preserved. Since the storage-ring components are not involved, pulse picking using MEMS can be set up at any hard X-ray beamline in a similar fashion as conventional mechanical choppers, but orders of magnitude faster and miniaturized.

In this work, using MEMS-device-based ultrafast X-ray optics operating at large oscillation amplitudes, we have obtained an unprecedented accomplishment in manipulating X-ray pulses on time scales of 300 ps. By utilizing the diffractive capability of a fast-oscillating MEMS device through Bragg angles for a monochromatic X-ray beam, a dynamic MEMS device can be an ultrafast X-ray pulse-picking instrument that maintains the spatiotemporal correlation of synchrotron X-rays with a 300-ps temporal resolution. This capability will be extremely useful at higher repetition rate, low-emittance X-ray sources worldwide. By developing ultrafast devices, we show the overwhelming synergy between the scalable MEMS-based dynamic optics and future X-ray sources. The immediate application of the devices is to create new timing structures from high-repetition-rate storage-ring-based sources that are currently not suitable for time domain sciences. Further capability entails multiplexing X-ray pulses for time-domain experiments at a synchrotron radiation or high-repetition-rate XFEL source, so that multiple experiments can be accommodated simultaneously at a single-beam facility. This application requires higher *f* (for example, 100 kHz for a 1-MHz XFEL source) but a moderate DTW. The effects of radiation damage may need mitigation before the device can be used as a practical multiplexer at an XFEL source, but we note that our devices have run continuously when illuminated by an unfiltered synchrotron white beam with a power density of ca. 100 W/mm^2^, modulated to a 1-Hz pulse train of 1.5% duty cycle (a macro-scale mechanical shutter opening 15 ms out of every 1 s). Looking beyond manipulating monochromatic X-rays, other applications include those that are currently accomplished by bulk X-ray optics such as a fast-scanning X-ray spectrometer or monochromator. Since the MEMS devices are almost 100% efficient, devices with such a narrow DTW can enable sub-nanosecond time-resolved research with lab-based X-ray sources.

## Methods

### Design and fabrication of MEMS

Drastically improved from the asynchronous MEMS used in our previous work, the P0/2 and P0 devices were designed to consist of a (001)-oriented silicon single crystal suspended by a pair of comb-drive torsional actuators. The dimensions of the crystal are 25 µm in thickness and 250 × 250 µm in the lateral directions. The comb-drive actuators are inter-digitated capacitors that provide electrostatic force to excite out-of-plane oscillation of the silicon crystal around the axis joining the torsional actuators. Beyond the region of oscillating silicon crystal, electric wires were laid out connecting comb-drive actuators to electric inputs. Finite element analysis and CoventorWare simulation were utilized to aid the design with calculation of the modal response and resonance frequency of the MEMS devices. The fabrication was carried out at the commercial foundry MEMSCAP employing the SOIMUMPS process^44^. The structural material is a silicon-on-insulator wafer that provides the single-crystal silicon oscillatory element necessary to diffract X-rays. We note that the SOIMUMPS fabrication process also includes steps of phosphorus doping and diffusion that introduce strains to the top silicon layer. The dopant is responsible for the shoulder peaks near and slightly above the Bragg reflection of silicon (slightly contracted lattice spacing).

### Tuning the MEMS resonance frequency using a focused ion beam

The tuning of the MEMS device resonance frequency was carried out using a focused ion beam (FEI Nova 600 NanoLab) housed inside a class-100 clean room at the Argonne Center for Nanoscale Materials. The device was grounded to the sample holder before loading into the sample chamber. The Ga ion source was tuned to 30 keV, 21 nA. A standard milling program for silicon was used to etch a set of rectangles away from the edges of the silicon crystal farthest from the oscillation axis of the torsional flexures, as shown in Fig. 2b. In each tuning step, the removed pieces were symmetric to the axis of oscillation in order to avoid introducing an undesirable modal response in the device.

### X-ray measurements

X-ray measurements were carried out at experiment station 7ID-C at the APS. The static X-ray measurements of the rocking curves of the MEMS devices were identical to those described previously^29^. For the time-resolved metrology of the MEMS devices, we used two APS X-ray timing modes: standard 24-bunch mode with a pulse interval of 153 ns, and 324-bunch mode with a pulse interval of 11.37 ns. In both cases, the X-ray pulses are evenly distributed in time as the storage-ring is operated at 271.555 kHz for a period of 3.68 µs. Incident X-rays were monochromatized to an energy of 8 keV using a diamond (111) double-crystal monochromator. The X-ray beam was focused to ~10-µm horizontally by a rhodium-coated mirror, and confined by beam-defining slits to a 10-µm spot vertically. The MEMS device was mounted on a six-circle diffractometer for high-precision angular and lateral positioning. The X-ray beam was aligned to the center of the single-crystal silicon mirror at the (004) Bragg reflection in the vertical plane, thus taking advantage of the low emittance of the APS X-ray beam in the vertical direction. The diffracted X-rays passed through a flight path in vacuum and were then detected with customized avalanche photodiodes (APDs). The pulse signal of the APD could be sent either to a scaler (Joerger VSC16) to acquire the X-ray intensity (counting mode), or to a high-speed digitizing oscilloscope (Yokogawa DLM4058) to record the real-time X-ray response (integrating mode).

### Measuring the X-ray DTW of a MEMS device via delay scans

To measure the sub-nanosecond DTW of the MEMS oscillators in real time, one would need a continuous X-ray source on the nanosecond time scale and an X-ray detector with 10-ps time resolution. The storage-ring frequency-matched MEMS devices allow us to measure the DTW using delay scans with synchrotron pulses of 100-ps FWHM and a detector with nanosecond time resolution. The delay scans are illustrated in an animation in Supplementary Note 4. When the delay between the oscillatory motion of the MEMS device and an X-ray pulse from the APS is adjusted with a 20-ps step, the response from the slow detector generates the DTW profile with 20-ps time resolution. The measurement normally takes a few tens of seconds to complete, which is orders of magnitude more efficient than the coincidental scan with asynchronous devices used in our previous work^29^.

### Measuring MEMS oscillation amplitude with X-ray pulses

The DTW of a MEMS device is inversely proportional to its oscillation amplitude, which is extremely sensitive to the environment’s temperature and pressure. Therefore, evaluating the MEMS oscillation amplitude using X-ray pulses is critical before or after the DTW measurement. However, the temporal delay scan is not applicable for measuring the oscillation amplitude measurement. With the previous asynchronous device, the oscillation amplitude was measured precisely with coincidental scans by recording MEMS-diffracted X-ray pulses using a fast detector and a digitizing oscilloscope. The measurement of the frequency-matched device was similar to the coincidental mode, but it required high-frequency (or high-repetition-rate) X-ray pulses. The detail is described in Supplementary Note 3 and we give only a brief account of the method here. When the MEMS resting angle (*θ*_0_) with respect to the X-ray beam is set to the Bragg angle (*θ*_B_), the diffracted pulse happens at time Δ*τ* = 0 (zero phase difference). If this angle is set to be slightly different from *θ*_B_, the MEMS will diffract an X-ray pulse when the MEMS rotates to Δ*θ* = *θ*_0_ − *θ*_B_ and the pulse coincidentally strikes the MEMS at the time instance (Δ*τ* ≠ 0, or a non-zero phase difference). A fit of Δ*θ* vs Δ*τ* results in an accurate oscillation profile and amplitude even if only a small segment of data around Δ*τ* = 0 is collected. Since the device is frequency-matched to the storage-ring or the incoming X-ray pulses, the coincidence-mode only works efficiently when the X-rays are densely populated in the time domain, so the 324-bunch mode of the APS was used. As the MEMS amplitude increases, its DTW width decreases and the probability of coincidence between the MEMS device’s diffractive window and the incoming X-ray pulses decreases as well.

## Supplementary information

Supplementary Information

Supplementary Movie 1

## Data Availability

The data that support the findings of this study are available from the corresponding author upon reasonable request.
