# Supplementary material for: Ultrafast photonic micro-systems to manipulate hard X-rays at 300 picoseconds
Source: Nat Commun. 2019 Mar 11;10:1158. doi: 10.1038/s41467-019-09077-1 (PMC6411987; doi:10.1038/s41467-019-09077-1)
Supplement: Supplementary file 1 — Supplementary Information [file 41467_2019_9077_MOESM1_ESM.docx]

**Ultrafast photonic micro-systems to manipulate hard x-rays at 300 picoseconds**

Pice Chen, et al.

**Supplementary Information**

**Supplementary Note 1. Tuning of the resonance frequency by focused ion beam machining**

The micro-electro-mechanical systems (MEMS) devices are torsional actuators with a resonant frequency *f* as^1^:

$f= \sqrt{\frac{k}{I}}$, (Eq. 1)

where *k* and *I* are the stiffness of the torsional flexure and the moment of inertia of the oscillating part. As in a rectangular prism, *I* = $\frac{1}{12}$*m*(*L*_eff_^2^ + *H^2^*) ≈ $\frac{1}{12}$ρ*HWL*_eff_^3^, where *m* and ρ are the mass and density of the structural material silicon; and *H* (thickness), *W* (width), and *L*_eff_(effective length)are the dimensions of the silicon mirror, as denoted in Supplementary Figures 1a and 1b. The FIB process milled away silicon from the outer edges of the mirror and resulted in notches as shown schematically in Supplementary Figures 1a and 1b. To simplify the calculation, we approximated the notched mirror as a rectangular prism using an effective length *L*_eff_. The change in the mass of the silicon mirror resulted from the FIB process is equivalent to the change of the effective length, which in turn shifts the resonant frequency. Taking the derivative of Supplementary Equation 1, we can deduce that the resonant frequency shift ($\Delta f$) is related to the change of mass ($\Delta m)$as

$\frac{\Delta f}{f}=-\frac{3\Delta m}{2m}.$ (Eq. 2)

Therefore, the increase of the resonance frequency is proportional to the amount of removed mass from the far edges of the element. An example of the shift of frequency of a P0/2 MEMS device during the FIB process is shown in Supplementary Figure 1c. To be consistent with the main text, the frequency here is referred to the driving frequency, twice the value of the resonance frequency. The target value of the driving frequency for this device is 135.777 kHz. The single-crystal element has a dimension of 250 μm by 250 μm by 25 μm, corresponding to a total mass of 3640 ng. Based on Supplementary Equation 2 we can calculate the expected slope to be 56 Hz/ng, in good agreement with the measurement, where the slope from the linear fit to the data is 49 Hz/ng.


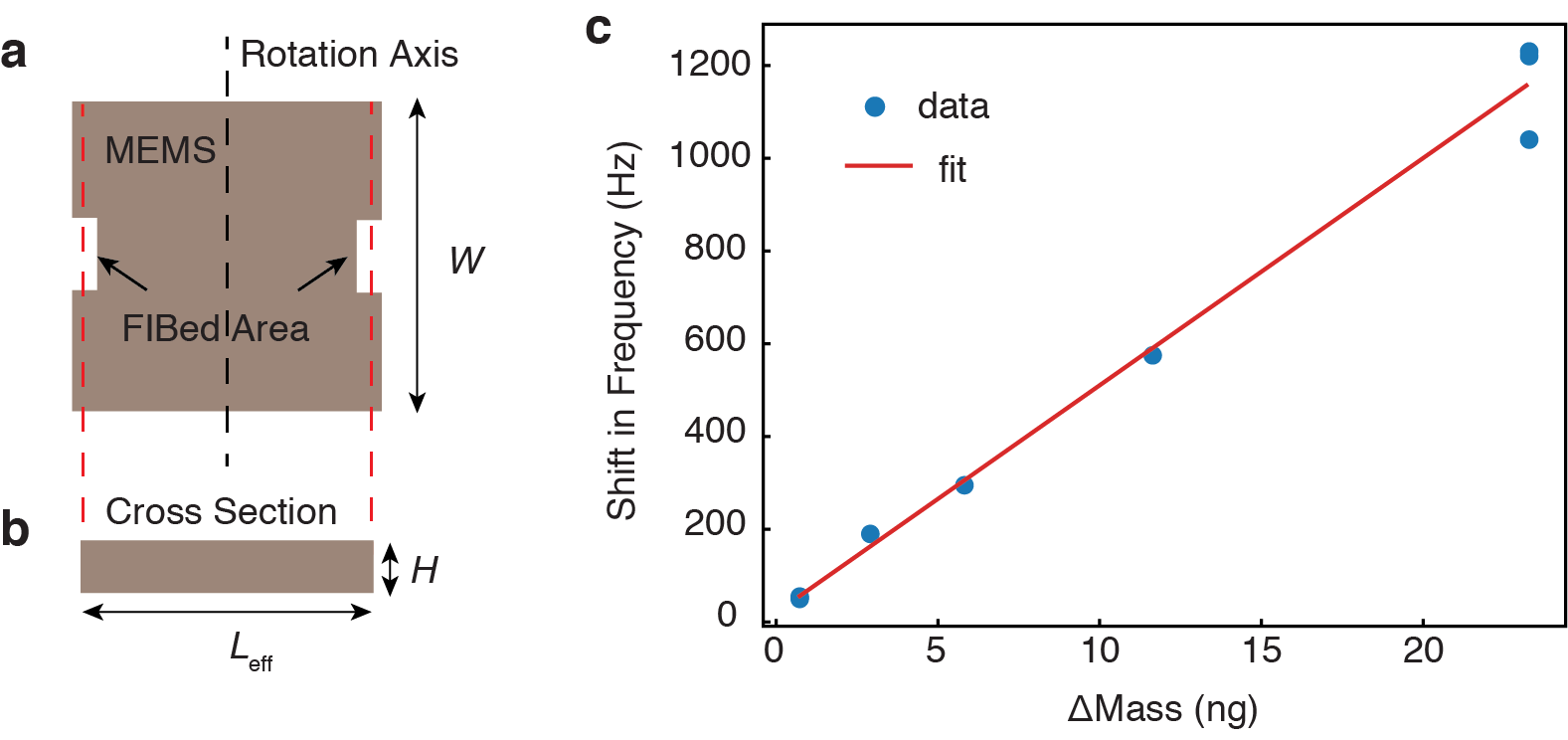


**Supplementary Figure 1.** Tuning the resonance frequency of MEMS devices by FIB machining**.** **a** Schematic of the x-ray diffracting silicon element of a MEMS device, after the FIB process symmetrically machined away the notched areas. **b** Schematic of the cross section of the silicon element that is approximated as rectangular. **c** The shift of the peak frequency of the tuning curve of a P0/2 MEMS device, as a function of the amount of the mass of silicon removed after each FIB process. The line is a linear fit to the data.

**Supplementary Note 2. Static rocking curve of MEMS devices**

The static rocking curve was measured when a MEMS device was not energized. The rocking curves of the frequency-matched devices were acquired with conventional *θ**θ* scans. We use a hard x-ray beam with a photon energy of 8 keV. The x-ray beam was focused horizontally and confined by a pair of slits to approximately 10 µm × 10 μm before impinging onto the center of the silicon resonator of the MEMS device. A high-precision diffractometer with an angular resolution of 3 × 10^-5^ degree was used, as the width of the rocking curves is on the order of a few millidegrees.


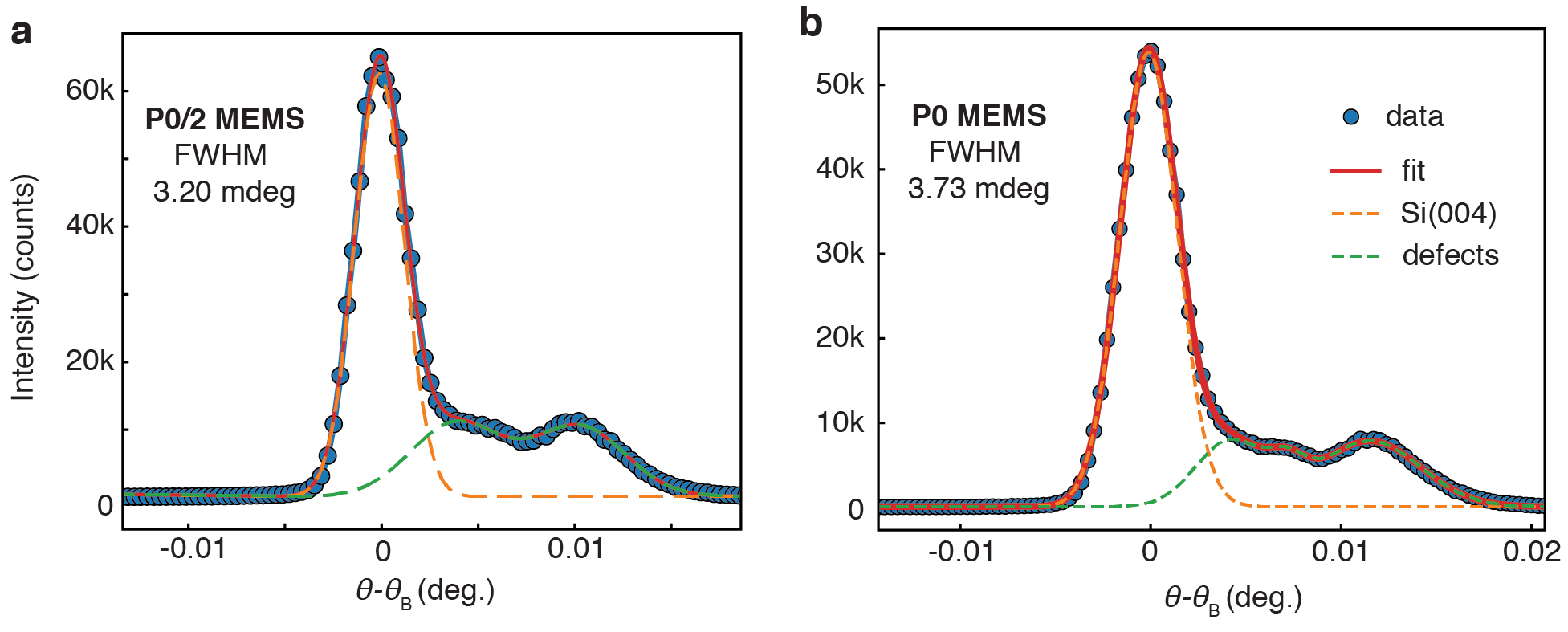


**Supplementary Figure 2.** Rocking curves of the silicon crystal resonator at the Si (004) Bragg reflection**.** **a** From a P0/2 MEMS device and **b** From a P0 device. The lines are fit to the data, with two components: diffraction from single-crystal silicon (004), and diffraction from phosphorous dopant-induced strained layers on the surface.

Supplementary Figure 2a shows the rocking curve for a typical P0/2 MEMS device measured around the (004) Bragg reflection of the x-ray diffracting silicon mirror. In addition to the strong Bragg reflection with a full width half maximum (FWHM) of 3.20 mdeg, the defects of the silicon mirror, particularly its doping layers induced during the fabrication process^2^, contribute to a high-angle shoulder. A similar observation of the side peaks has been reported in our previous work on asynchronous MEMS devices.^3^

By far our fastest devices are the P0 MEMS devices, with a resonance frequency twice that of the P0/2 devices. In term of the static structure, however, the devices are similar to lower-frequency devices including the asynchronous ones, as all these devices were fabricated using the same fabrication process. A typical rocking curve is shown in Supplementary Figure 2b, consisting of a 3.73-mdeg Bragg reflection as well as side peaks at higher angles. We should note that the side peaks contribute to all the dynamic rocking curve profiles measured in the time domain shown throughout this work.

**Supplementary Note 3. Measurement of oscillation amplitude with x-ray pulses**

In our previous work, we demonstrated that x-ray pulses from the Advanced Photon Source (APS) storage ring can be used to measure the oscillation amplitude of asynchronous MEMS devices by recording the MEMS diffraction wave form^3^. As shown in Supplementary Figure 3a, similar metrology can be performed with the frequency-matched MEMS device using high-repetition x-ray pulses, such as those of the 324-bunch mode at the APS. We operate the MEMS device around the Bragg condition with an oscillation angle $\theta_{\mathrm{MEMS}}=\theta_{0}+\alpha sin(\omega t)$, where *θ*_0_ is the x-ray incident angle when the silicon element is at its rest position, *α* and $\omega$ (or *2π/T*) are the oscillation amplitude and angular frequency, respectively. As illustrated in Supplementary Figure 3b, the Bragg condition is met twice in one oscillation cycle, but the separation between these two times depends on *θ*_0_ of the MEMS device. For example, when *θ*_0_*=*__, the separation is half of the cycle, and this is the reason that a MEMS device can pick x-ray pulses at a frequency twice its resonant frequency. When *θ*_0_ *≠*__* * the separation alternates between less than and greater than half of the cycle.


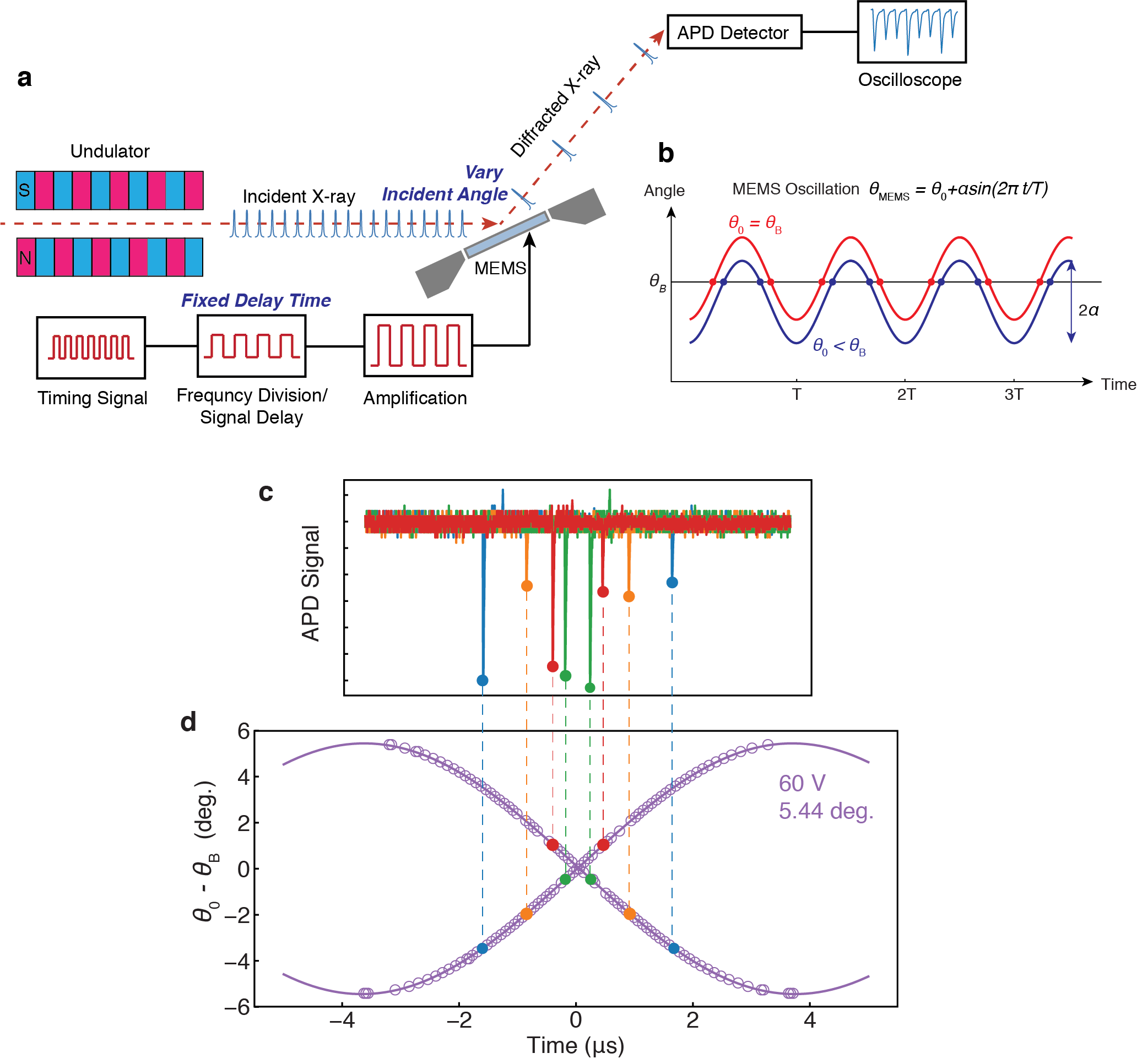


**Supplementary Figure 3.** At-wavelength measurement of MEMS resonator oscillation amplitude**.** **a** Schematic of experimental setup using x-rays from 324-bunch mode at the APS. **b** Schematic showing the relationship between Bragg angle (*θ*__) and the oscillation angle of MEMS devices (*θ*_MEMS_). The dots mark the times when the oscillation angle is equal to the Bragg angle, or when the Bragg condition is satisfied during oscillation. **c** Scope traces of real-time x-ray response to an oscillating MEMS device (P0/2, excited with 60 V). The time-axis is folded so that each half-cycle measurement is overlaid onto the other within the full cycle. Different colors represent the measurements at different resting theta positions *θ*_0_ of the MEMS device. **d** Resting theta angle, with respect to Bragg angle, plotted as a function of the relative times where x-ray pulses were observed in the scope traces. The two lines are sinusoidal fits to the angular rotation of MEMS device.

To measure the oscillation amplitude, we collected a series of scope traces of diffracted x-ray pulses (negative voltage pulse from the avalanche photodiode) in real time when the resting position of MEMS devices is varied over a wide range, typically *θ*_B_ ± 5 degrees. These scope traces are plotted by overlapping the clockwise half-cycles of a MEMS device on top of the counterclockwise ones (Supplementary Figure 3c). Peaks in the scope traces represent the instances when an x-ray pulse impinges onto the MEMS element at the Bragg condition. Since the relative position of the peaks depends on the *θ*_0_ of the MEMS device, as mentioned above, the correlation between the peak positions and theta angles yields the oscillation trace $\theta_{\mathrm{MEMS}}=\theta_{0}+\alpha sin(\omega t)$. Supplementary Figure 3d shows *θ*_0_ of MEMS devices plotted against the relative times where x-ray pulses are picked, thus mapping out two branches of the sinusoidal oscillation of MEMS devices. A sinusoidal fit, with known oscillation frequency, determines the exact oscillation amplitude. In this example, shown in Supplementary Figures 3c and 3d, a P0/2 MEMS device, operated at 60 V, has an oscillation amplitude of 5.44 degrees.

**Supplementary Note 4. Delay scan for measuring a diffractive time window**

Intuitively, a diffractive time window (DTW) can be measured with a continuous x-ray beam using an x-ray detector with a temporal resolution much shorter than the DTW width. Such high-temporal-resolution x-ray detectors are not readily available. Since an x-ray beam from a storage ring source is always pulsed and the pulse width is shorter than current DTWs, one can use temporal delay scans to effectively map out the latter. The delay scans become efficient especially when the MEMS oscillation is frequency-matched to the storage ring so that we can achieve high temporal resolution without recourse to a fast x-ray detector. This concept of mapping the DTW is illustrated in Supplementary Movie 1, as the oscillation of MEMS device is frequency-matched to a sub-frequency of x-ray pulses from storage ring at 88 MHz (11.4-ns pulse interval, 324-bunch mode at APS). When the phase difference between the MEMS oscillator and an x-ray pulse is varied, the particular x-ray pulse from the storage ring samples through the DTW of the MEMS device element.


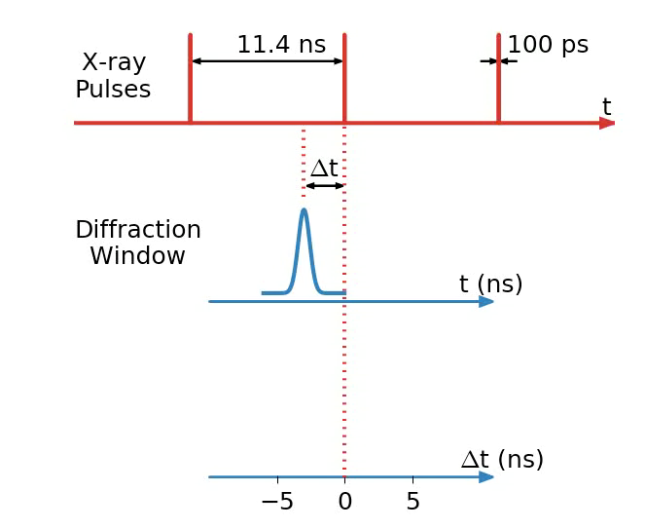


**Supplementary Movie 1**. Concept of mapping diffractive time window with delay scans.

Experimentally, the measurement was performed using the setup shown in Supplementary Figure 4a, at beamline 7-ID of the APS. Since the exciting voltage pulse for the MEMS is synchronized to the storage ring signal, the phase of the MEMS oscillation can be adjusted with a delay generator in the driving circuit. To measure a DTW profile, the resting position of the MEMS device is set at the Bragg angle respective to the x-ray beam. Once the MEMS device starts to oscillate, the Bragg condition is met twice in one cycle: one each during the first and the second half-cycle. These two dynamic rocking curves in the time domain have opposite waveforms since the MEMS element rotates in opposite directions across the Bragg conditions. The rocking curves in the time domain are recorded by a counting detector, which does not need to be particularly fast (Supplementary Figure 4a); in our case, it is a customized avalanche photo diode detector. When the resting position of the MEMS device is set at the crystal Bragg angle, the two dynamic rocking curves are separated by exactly half of the oscillation cycle, as illustrated in Supplementary Figure 4b. With a zero delay time, when the first dynamic Bragg peak in the cycle coincides with an x-ray pulse, the second dynamic Bragg peak will coincide with another x-ray pulse half an oscillation cycle away, as shown in Supplementary Figure 4b. By stepping through a series of delay times, a pair of x-ray pulses map out the entire time-domain rocking curve (or DTW). Since the detecting circuit cannot discriminate between the two rocking curves in one oscillation cycle, a scanned rocking curve shows “mirror-imaged” features (Supplementary Figure 4c) from the static rocking curve, including the dopant-induced strain peaks above the Bragg peak at later (earlier) times for increasing (decreasing) angles.


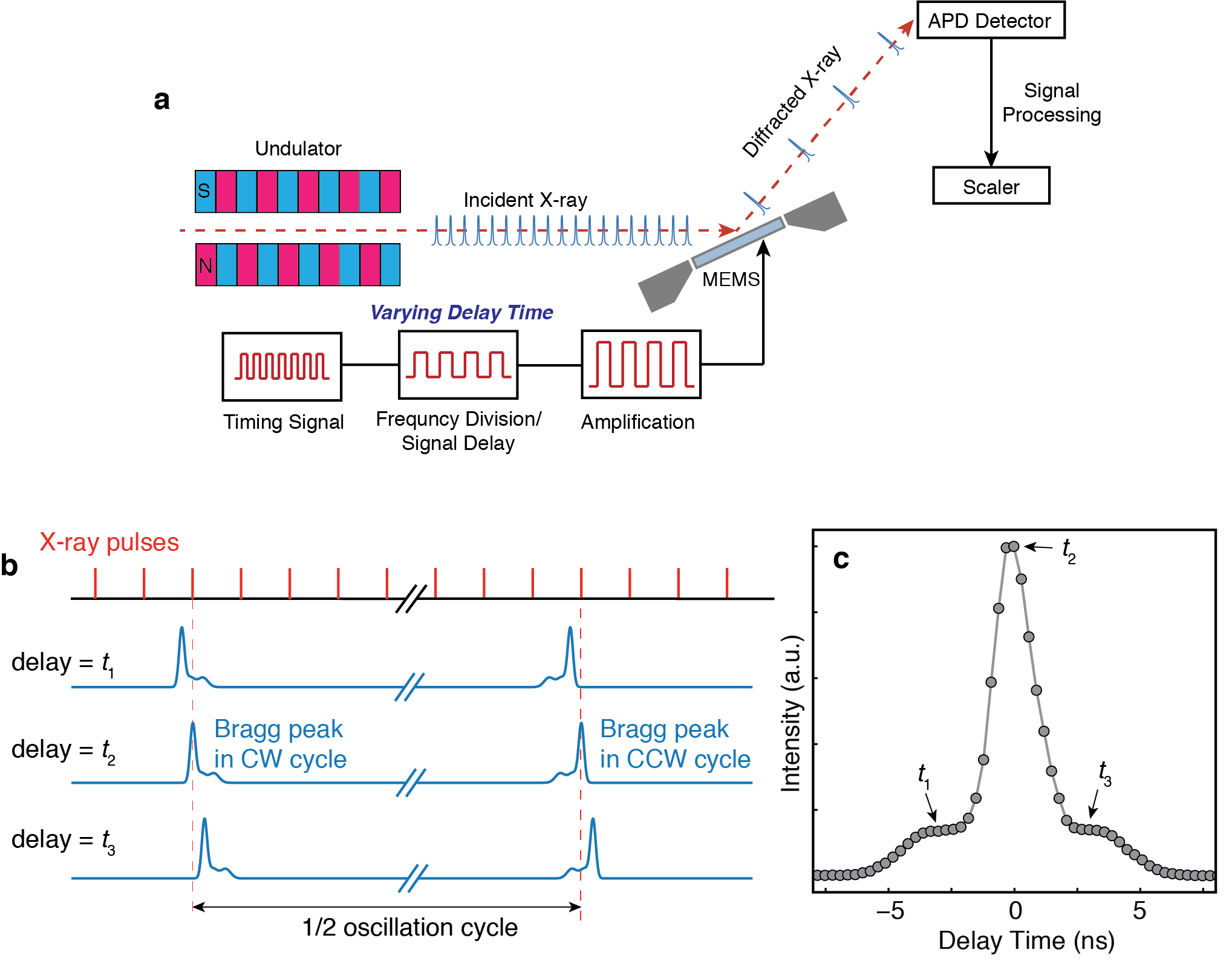


**Supplementary Figure 4.** Measuring the dynamic rocking curve by delay scans. **a** Schematic of the experimental setup used to perform delay scans. **b** Schematics of periodic x-ray pulses from a storage-ring synchrotron source, as well as Bragg peaks of MEMS devices in time domain, at three different delay times (*t*_1_ < 0, *t*_2_ = 0, *t*_3_ > 0) where the zero-delay-time is when the peak of the dynamic rocking curve coincides with one of the x-ray pulses. **c** Example delay scan mapping out the diffractive time window of a MEMS device. The effect from the side peaks of the dopant layer is apparent on both sides of the (400) Bragg peak because the peaks are scanned from both angular directions. *t*_1_, *t*_2_, and *t*_3_ are the three delay time values illustrated in **b**.

**Supplementary Note 5.** **Profile of an x-ray beam diffracted by MEMS devices**

With frequency-matched MEMS devices, a sub-nanosecond DTW for temporal manipulation of synchrotron x-ray pulses is achieved while maintaining the spatial profile of incident x-ray beam when the device is rapidly oscillating. This is demonstrated as follows. The sample was mounted on a 6-circle diffractometer where the sample-to-detector distance was 1.10 m. We performed 2-theta scans with a fine slit (10 µm) to profile the incident beam (without the presence of the MEMS device) and the diffracted beams when the MEMS was static at the Bragg angle or oscillating about the Bragg angle. Supplementary Figure 5 shows the beam-profile measurements by scanning the detector with the fine slit. The incident x-ray beam, confined to 6 μm by beam-defining slits located 20-cm upstream of the diffractometer, was enlarged to about 27 μm FWHM due to coherent scattering from the slits. In comparison, the x-ray beam diffracted off the surface of the static MEMS (Supplementary Figure 5b) was measured to be 48 μm FWHM using a 2-theta scan. This broadening was primarily due to the presence of a doped layer that introduced defects in the x-ray diffracting MEMS element, as discussed above in Supplementary Note2. We expect that the diffracted beam will be further improved with modified fabrication methods removing the doping layers. When the device was turned on and the silicon mirror oscillated rapidly, the beam size became sensitive to distortions of the MEMS element at an angular speed > 10^6^ degrees s^-1^ as well as any timing jitter in the system. It is thus remarkable that the diffracted beam was only slightly broadened to 63 μm in the oscillating case (Supplementary Figure 5c). These measurements confirm that the MEMS devices serving as x-ray optics can pass the x-ray beam profile downstream with a profile acceptable for practical x-ray studies.


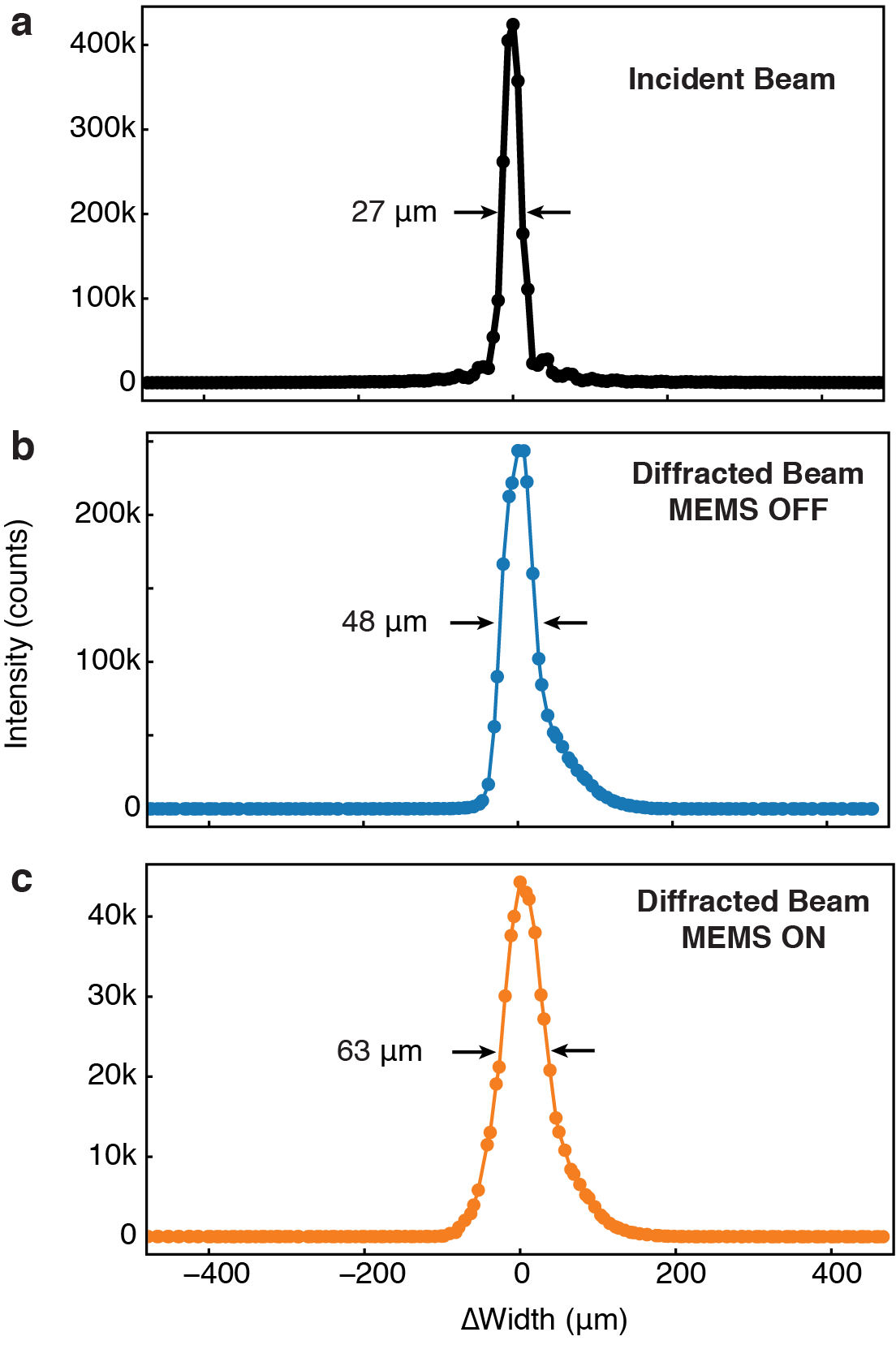


**Supplementary Figure 5.** Measurement of the beam spatial profile. **a** Incident beam profile and diffracted beam profile measured when the MEMS device is **b** stationary and **c** oscillating. The measurement was conducted by scanning the angle of the detector arm, then converting the angular position to spatial units for better representation of beam size.

**Supplementary Note 6.** **Stability of devices under synchrotron x-ray beam**

The MEMS devices are designed to manipulate synchrotron x-rays and are required to sustain significant x-ray dose without degradation of their performance. At current and future synchrotron sources, the intensity of a monochromatic, collimated beam on the order of 10^11^ photon/s between 8 to 14.4 keV will result in 23 to 61 µW of absorbed power, as shown in Supplementary Table 1. Thanks to the excellent thermal conductivity of silicon, our MEMS devices will maintain their structural and mechanical characteristics under this level of x-ray dose. Neglecting convection and other potential sources of heat transfer, if the energy deposited by the x rays is transported out of the rotating MEMS crystal solely by conduction through the torsional springs (using device dimensions described below in Supplementary Note7), the upper limit of the steady-state temperature rise is calculated to be about 0.28 K for a beam flux of 10^11^ photons/s at 8 keV.

| **Supplementary Table 1** Estimate of x-ray absorbed powerby a MEMS device. The calculation was performed in the vicinity of the Bragg condition for an incident beam with a flux of 10^11^ photon/s. | | | | |
| --- | --- | --- | --- | --- |
|  | Energy (keV) | | | |
|  | 8.0 | 10.0 | 12.0 | 14.4 |
|  (Å^-1^) | 1.55 | 1.20 | 1.01 | 0.861 |
| Effective thickness (µm) | 45.3 | 56.6 | 67.9 | 78.9 |
| Absorption | 0.478 | 0.345 | 0.257 | 0.182 |
| Absorbed Power (µW) | 61.2 | 55.2 | 49.3 | 23.3 |

We demonstrate the thermal stability of the MEMS devices under monochromatic hard x-rays. For these measurements, we used an x-ray beam with flux of the order 10^10^ photons/s at 8 keV (approximately 6 µW of absorbed power). Two measurements were conducted. First, we monitored the diffracted intensity at the middle point of the rising edge of the rocking curve, where it is most sensitive to peak shift. If x-ray beam-induced heating were significant, it would cause lattice expansion, shift the peak to lower angles, and increase the intensity at the shoulder. Supplementary Figure 6a shows that after the x-ray shutter was opened at *t* = 0, the intensity at the shoulder remained constant over 50 s without an intensity increase.

In the second experiment, we acquired a series of static rocking curves to monitor the peak position after the x-ray shutter was opened at *t* = 0. The curves shown in Supplementary Figure 6b display relative peak shifts for two series of measurements, one scanning *θ* low to high and the other high to low, to guard against systematic errors. The peak shifts in Supplementary Figure 6b are negligible within the experimental resolution and show no measurable heating trend. Specifically, the average peak shift is 0.024±0.024 mdeg at time 50-150 s, and -0.004±0.02 mdeg at time 410-520 s. For reference, a 1-K temperature rise corresponds to approximately a -0.1 mdeg shift. Similar measurements also showed no measurable heating at lower x-ray fluxes.

*
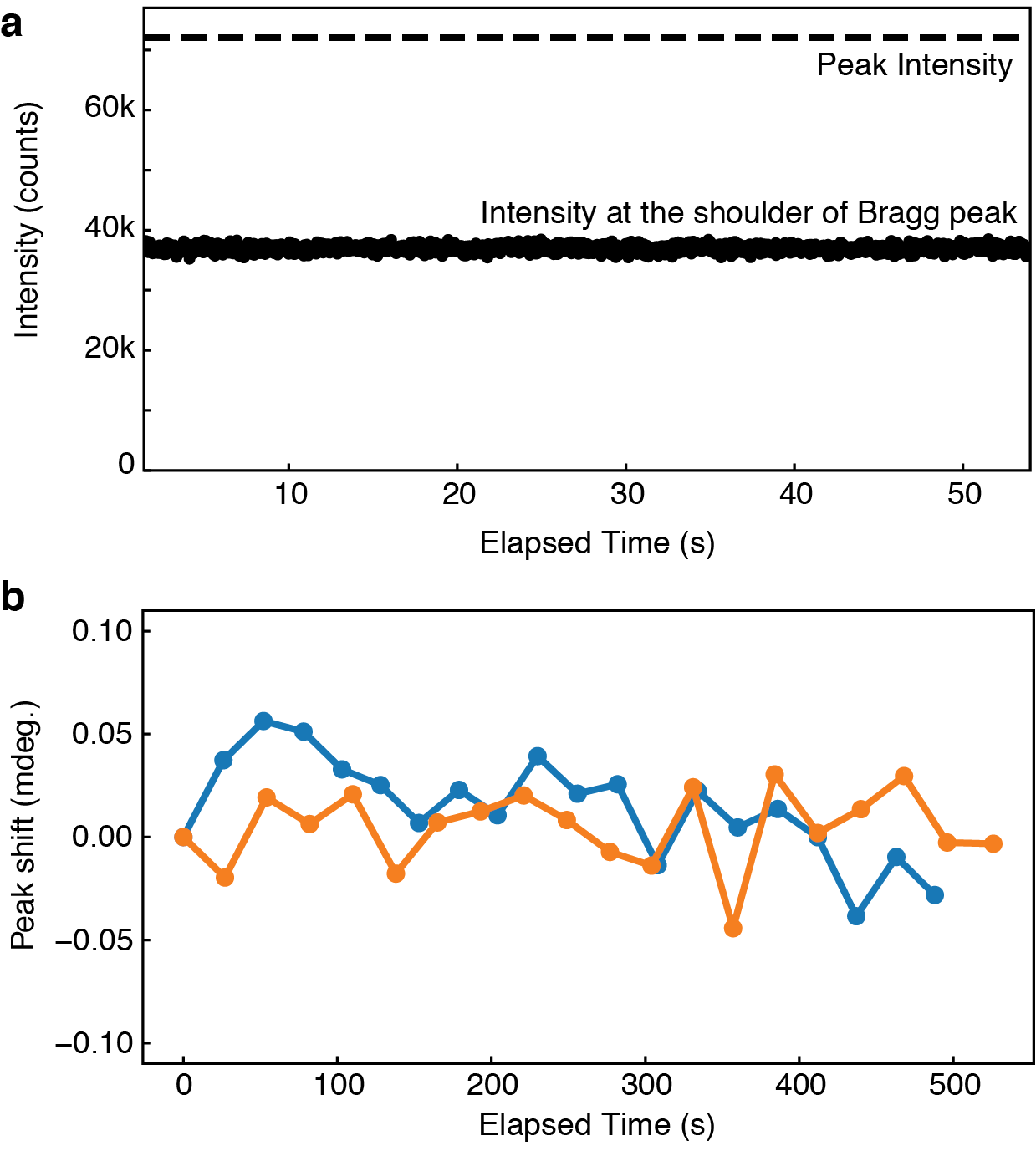
*

**Supplementary Figure 6.** Thermal stability of MEMS devices under monochromatic hard x-rays. **a** Intensity at the low-angle shoulder of Bragg peak as a function of time, after x-ray turned on at *t* = 0. X-ray flux in this measurement is estimated to be 2×10^10^ photons/s. **b** Relative shift of peak position of rocking curves of MEMS device. The plots are from two series of scans, one scanning  low to high (blue) and the other high to low (orange).

In addition to addressing the beam heating effect under monochromatic x-ray beam, we have also investigated the stability of a MEMS device under a much higher x-ray dose using white-beam measurement. The measurement was carried out in x-ray imaging beamline at 7ID-B of APS. The incident white x-ray beam has a power of about 400 W and a center photon energy of 11.6 keV. A mechanical shutter, which opens 15 ms in every 1s, was used to modulate the white beam and cut off 98.5% of the incident power. Also considering that the white beam (1 mm × 1 mm) is much larger the MEMS resonator, we estimate that the power of x-ray beam impinged on the device is on the order of 10 mW, two orders of magnitude higher than the monochromatic case discussed above. We observed that over the course of several hours, the device can still operate continuously without apparent degradation under the 10 mW absorbed power. Regard the application of MEMS devices at high-rate FEL sources, we expect similar modulation devices are required to limit a long exposure of devices to FEL beam.

**Supplementary Note 7. Estimation of maximum deflection angle before fracture failure**

As seen in the MEMS device frequency response and oscillation amplitudes vs. driving voltage, the dynamic amplitudes show linear increases in amplitude even though static displacement vs. voltage is a quadratic relationship. This can be attributed to the increased dynamic damping of the device at larger velocities with increased oscillation amplitude. Although the MEMS device can be driven to very large amplitudes, resulting in stresses approaching and beyond the silicon yield strength at the flexures, our devices ultimately stop oscillating before failure, at voltages above 100 V. This is likely due to the fundamental mode of operation being unsustainable due to the large forces applied on the comb drives, which cannot be supported by the dynamically deforming flexures.

We estimated the angle at which the MEMS device is subject to fracture failure. As shown in the drawing in Supplementary Figure 7, the Si crystal, serving as an x-ray diffraction element, is connected to two sets of comb drives with a set of torsional springs. One end of the torsional spring is anchored at 0° by the frame. At a rotation angle of *θ*, the end of the torsional spring connecting to the Si crystal is thus twisted by an angle of*  * In this case, the torsional spring has the largest mechanical stress and will be the first part to fail.


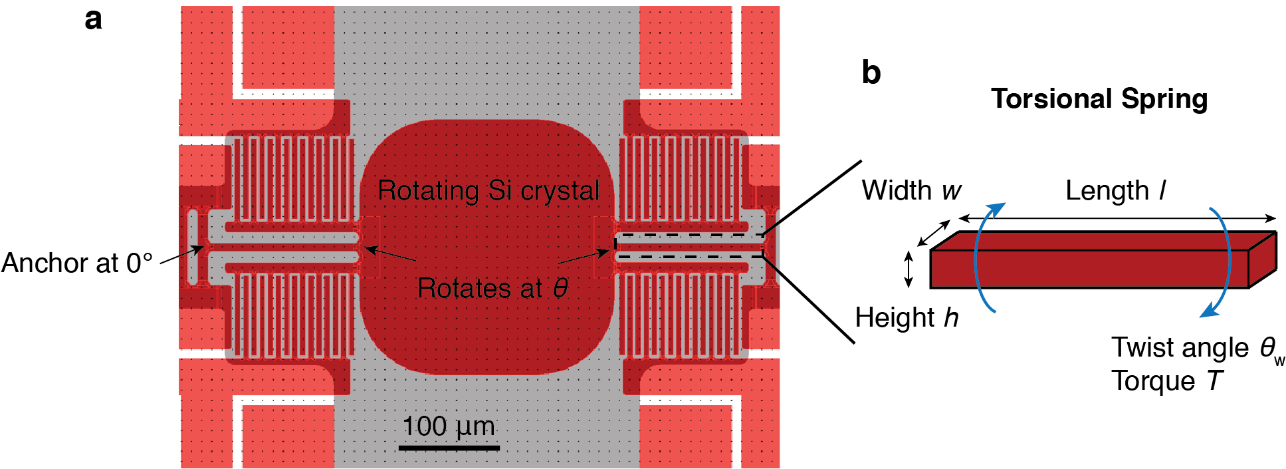


**Supplementary Figure 7.** Model of fracture failure of MEMS devices. **a** Schematic of a P0/2 MEMS device where the mechanical boundary of the torsional spring (boxed region) is specified. **b** Magnified view of the torsional spring as a bar subjected to a torque *T* at both ends.

We modeled the torsional spring as a homogenous silicon bar under a torque *T* acting normal to the axis of the bar at both ends. The dimension of the spring is 5 μm in width *w*, 25 μm in height *h*, and 150 μm in length *l*. Both ends of the silicon bar are twisted by *θ*_w_, but along opposite directions. The twist angle *θ*_w_ is half of the rotation angle * *of the rotating Si crystal, so that the mechanical boundary is equivalent to what is assumed above. The shear stress *τ* at a distance *r* from the axis of rotation is:

$\tau=\frac{Tr}{J}=\frac{G\theta_{w}r}{l}, and T= \frac{G\theta_{w}J}{l},$ (Eq. 3)

where *J* is the torsional constant or the second moment of inertia, and *G* is the shear modulus. The maximum shear stress is at the outermost surface of the mid-point of the torsional spring, where *r* reaches maximum. The spring fails when the maximum shear stress reaches or exceeds the fracture strength *σ*_fr_ of silicon. Rearranging Supplementary Equation 3 gives a maximum twist angle as:

$\theta_{\max} = \frac{l \sigma_{\mathrm{fr}}}{Gr_{\max}}$ . (Eq. 4)

For the rectangular-shape spring, *r*_max_ is 12.75 μm, and the shear modulus of [001]-orientated silicon is 79.6 GPa^4^. The fracture strength for single-crystal silicon is usually quoted as 6.9 GPa^5^, however a more practical value is in the range of 2.0-3.0 GPa measured *in operando* in similar designs of MEMS devices^6,7^. Using a fracture strength of 2.5 GPa, we estimate the maximum twist angle for our MEMS devices is 21.1°, corresponding to a maximum oscillation amplitude of 42.2°.

**Supplementary References**

1 Evoy, S. *et al.* Nanofabrication and Electrostatic Operation of Single-Crystal Silicon Paddle Oscillators. *J. Appl. Phys.* **86**, 6072-6077 (1999).

2 Cowen, A., Hames, G., Monk, D., Wilcenski, S. & Hardy, B. *SOIMUMPs Design Handbook*. (MEMSCAP Inc., 2011).

3 Mukhopadhyay, D. *et al.* X-Ray Photonic Microsystems for the Manipulation of Synchrotron Light. *Nat. Commun.* **6**, 7057 (2015).

4 Hopcroft, M. A., Nix, W. D. & Kenny, T. W. What Is the Young's Modulus of Silicon? *J. Microelectromech. Syst.* **19**, 229-238 (2010).

5 Petersen, K. Silicon as a Mechanical Material. *Proc. IEEE* **70**, 420 (1982).

6 Wolter, A., Schenk, H., Korth, H. & Lakner, H. Torsional Stress, Fatigue and Fracture Strength in Silicon Hinges of a Micro Scanning Mirror. *Proc. SPIE* **5343**, 176-186 (2003).

7 Dickensheets, D. L. *et al.* Fracture Strength of SOI Springs in MEMS Micromirrors. *Proc. SPIE* **6466**, 64660E (2007).
